# Coexisting growing teratoma syndrome and gliomatosis peritonei following ovarian immature teratoma: a case report and literature review

**DOI:** 10.55730/1300-0144.5899

**Published:** 2024-10-07

**Authors:** Osman TÜRKMEN, Serra AKAR İNAN, Serap AKBAY, Mohammad İbrahim HALİLZADE, İnci HALİLZADE, Özlem MORALOĞLU TEKİN

**Affiliations:** 1 Division of Gynecologic Oncology, Department of Obstetrics and Gynecology, Faculty of Medicine, Ankara Yıldırım Beyazıt University, Ankara, Turkiye; 2 Division of Gynecologic Oncology, Department of Obstetrics and Gynecology, Faculty of Medicine, University of Health Sciences, Ankara, Turkiye; 3 Department of Pathology, Ankara Bilkent City Hospital, Ankara, Turkiye; 4 Department of Obstetrics and Gynecology, Ankara Bilkent City Hospital, Ankara, Turkiye; 5 Department of Obstetrics and Gynecology, Faculty of Medicine, University of Health Sciences, Ankara, Turkiye

**Keywords:** Growing teratoma syndrome, gliomatosis peritonei, ovarian immature teratoma

## Abstract

Growing teratoma syndrome (GTS) is characterized by a reduction in serum tumor markers despite the growth of a benign mature teratomatous mass following chemotherapy for germ cell tumors. Gliomatosis peritonei (GP) typically accompanies ovarian teratomas, marked by the dissemination of mature glial tissue across the peritoneum. The concurrent presence of GTS and GP after treatment for ovarian immature teratoma (IMT) is notably rare, with approximately 20 reported cases.

This case involves a 25-year-old patient who underwent surgical removal of an adnexal mass, which was later diagnosed as stage IIIA grade 3 ovarian IMT with parametrial involvement. Following two cycles of bleomycin, etoposide, and cisplatin chemotherapy, imaging identified new lesions adjacent to the liver and on the pelvic peritoneum. A second fertility-sparing surgery was performed, and paraffin pathology confirmed a mature teratoma within the excised specimen. Additionally, the resected pelvic peritoneum revealed nodules of mature glial tissue consistent with GP.

The coexistence of GP with GTS post-IMT surgery presents a diagnostic challenge in distinguishing between malignant and benign components, which is critical to avoid unnecessarily aggressive surgical and chemotherapeutic treatments. Recognizing such cases may enable fertility-sparing surgery for these patients.

## 1. Introduction

Growing teratoma syndrome (GTS) is an uncommon condition associated with ovarian immature teratoma, characterized by the paradoxical growth of benign teratoma tissues during or after chemotherapy for malignant germ cell tumors. Clinically, GTS often presents with an increasing abdominal mass or discomfort despite normalization of serum tumor markers like alpha-fetoprotein (AFP). GTS is a rare but significant entity associated more commonly with nonseminomatous germ cell tumors (NSGCTs) in males, but it can also be seen in ovarian germ cell tumors, most frequently in ovarian immature teratoma (IMT). Based on published cases [1–4], it is estimated to occur in 13%–20% of patients with IMT, but it has been reported to be as low as 2.6% [5]. GTS poses a diagnostic challenge as it can be mistaken for disease progression or recurrence due to its imaging characteristics, which include the presence of solid and cystic components.

Gliomatosis peritonei (GP) is another rare but related condition often associated with ovarian teratomas, both mature and immature. It involves the spread of glial tissue with mature components throughout the peritoneum [1,5]. Clinically, GP can present with nonspecific symptoms, such as abdominal pain or masses, and it is seen in a minority of cases involving IMT. Its exact incidence is not well documented in large-scale studies [5]. Although usually benign, thorough histopathological evaluation to rule out any malignant transformation is required in cases of GP.

The association of GP with GTS following treatment for ovarian IMT is particularly rare, with about 20 cases reported thus far. The coexistence of GP with GTS after ovarian IMT surgery poses a major challenge for the exclusion of malignancy, which is crucial for avoiding unnecessarily aggressive treatments. Conservative surgery may then be tailored for the management of such patients.

## 2. Case

A 25-year-old female patient (gravidity 0) presented with abdominal pain in February 2023. Her medical and family histories were unremarkable. The blood workup showed three elevated serum tumor markers, AFP at 2643 μg/L (normal < 8 μg/L), CA-125 at 145 U/mL (normal < 30 U/mL), and CA-19-9 at 113 U/mL (normal < 31 U/mL); the serum tumor marker hCG was normal at 2 mIU/mL (normal < 10 mIU/mL). An abdominal ultrasound revealed a 90 × 50 mm solid mass lesion containing calcifications and cystic growth, suspicious for an immature teratoma. The mass was located anterior to the uterus. Doppler ultrasound showed increased central vascularity of the lesion. Contrast-enhanced abdominal computed tomography (CT) showed a lobulated mass, likely a teratoma or dysgerminoma, which was noted anterior to the uterus. This mass extended towards the superior pelvic region, measuring up to 75 mm on the right and 65 mm on the left at its widest points. It contained areas of fat and calcification and appeared heterogeneous and hypodense. No evidence of metastases was seen in the abdomen or on contrast-enhanced thorax CT.

One week later, in February 2023, the patient underwent a laparotomy. A multilobulated mass with irregular surfaces, originating from the right adnexa and measuring approximately 13 × 16 cm, was observed. The right unilateral salpingo-oophorectomy specimen was sent for frozen section analysis, which indicated an immature teratoma. A systematic complete pelvic and paraaortic lymphadenectomy was performed from the level of the deep circumflex iliac vein to the renal vein. During the lymph node dissection, the left ovarian artery was preserved. A necrotic mass that was adherent to the uterine artery and ureter was observed just above the level of the right sacrouterine ligament. The mass was excised while preserving the ureter and the uterine artery. Several implants were observed on the omentum, and an omentectomy was performed. Palpation and observation of the abdomen revealed no other suspicious lesions.

On paraffin pathology, the tumor measured 17 cm in diameter and consisted of approximately 50% immature elements with a histological grade of 3. The capsule was macroscopically intact. No lymphovascular or perineural invasion was noted. The posterior parametrium (right sacrouterine ligament) was infiltrated with the immature teratoma. The collected lymph nodes were nonmetastatic. The ascitic fluid was negative for malignancy. The patient was deemed to have stage IIIA grade 3 immature teratoma with parametrial involvement.

Her postoperative course was uneventful. Three weeks later in March 2023, she underwent oocyte retrieval and cryopreservation. One month after surgery, her serum AFP level had dropped (67 μg/L; normal <8 μg/L), her CA-125 was 193 U/mL (normal <30 U/mL), her CA-19-9 was 57 U/mL (normal <31 U/mL), and her serum hCG was 43 mIU/mL (normal <10 mIU/mL). She received two cycles of bleomycin, etoposide, and cisplatin (BEP) chemotherapy, which were completed in April 2023. Her serum AFP and hCG levels normalized after the chemotherapy.

**Figure 1:**
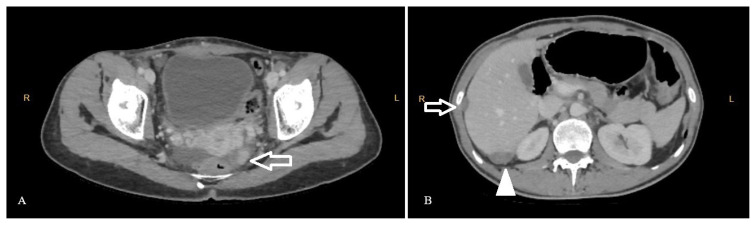
Axial view of the patient’s abdominal CT after primary surgery and chemotherapy. Contrast enhancing nodular implants are seen in the left pelvic peritoneum (Figure A, arrow). Figure B shows a 13-mm hypodense implant with punctuate calcifications causing indentation between liver segments 5 and 6 (arrow) and a 25-mm heterogenous hypodense lesion with calcifications on Morrison’s pouch (arrowhead).

However, the patient presented with generalized abdominal pain in May 2023. The contrast-enhanced abdominal CT scan in May 2023 revealed a dense lesion approximately 12 × 13 mm in size showing nodular enhancement consistent with a tumor deposit on the left pelvic peritoneal surface (Figure 1A). In the left adnexal area, dense solid structures consistent with implants or metastatic lymphadenopathy were identified adjacent to dilated pelvic vascular structures, where differentiation between them was not always clear. Additionally, right upper quadrant metastatic lesions approximately 10 × 16 mm in size were seen on the parietal and visceral peritoneal surfaces causing external indentation on the liver (Figure 1B). At the junction of the 5th and 6th hepatic segments posteriorly, there was an ovoid-shaped heterogenous hypodense lesion measuring 13 × 26 mm, causing mild indentation, and containing scattered millimetric punctate calcifications (Figure 1B). These imaging features were concerning for metastatic recurrence or residual tumor, which often demonstrate irregular contouring on imaging. However, these lesions were well circumscribed on imaging and the patient’s serum AFP, CA-125, and hCG were within normal range.

The patient had another operation in June 2023 due to a suspected recurrence in the peritoneum. During exploration, approximately 1.5-cm tumoral implants and a 2.5-cm lobulated mass were observed on the peritoneum adjacent to the liver and in the hepatorenal pouch. The observed masses were excised and sent for frozen section analysis. The frozen section results were consistent with mature teratomas. A 1-cm tumoral lesion adjacent to the left ovary peritoneum was excised. Millimetric lesions consistent with implants were observed on the bladder peritoneum and in the rectovaginal pouch. The peritoneum of the bladder and the rectovaginal pouch were excised. The uterus and the left ovary were conserved.

**Figure 2:**
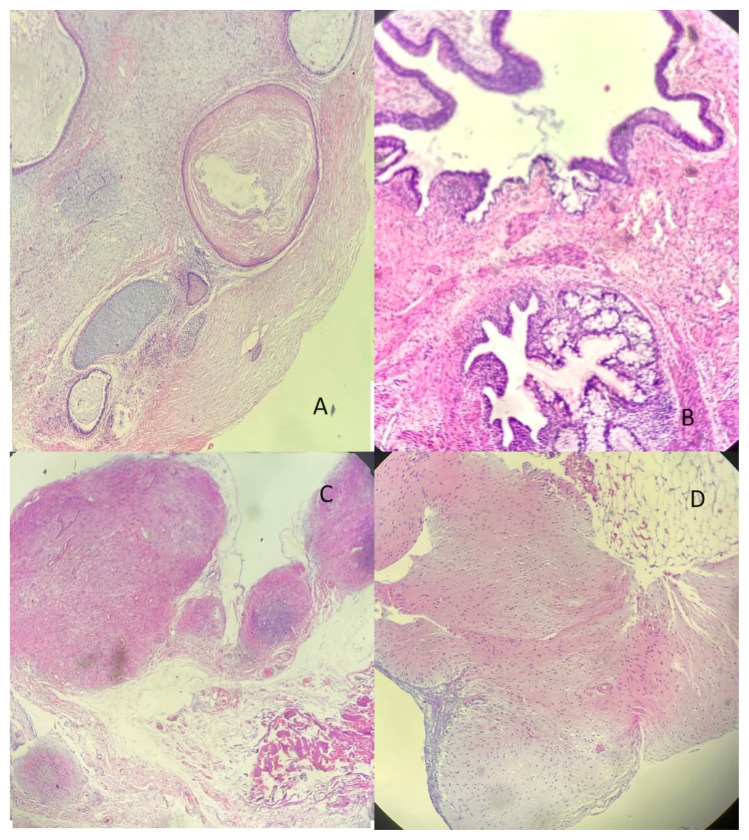
Paraffin pathology of the patient following the second surgery confirm (A) a mature teratoma within the hepatorenal pouch peritoneum (hematoxylin eosin staining, 4×), (B) mature teratoma cells without any immature components (hematoxylin eosin staining, 10×), (C) gliomatosis peritonei (hematoxylin eosin staining, 4×), and (D) mature glial cells seen within the resected pelvic peritoneum (hematoxylin eosin staining, 10×).

Paraffin examination of the specimens from the right diaphragm, hepatorenal pouch, rectovaginal pouch, and bladder peritoneum revealed multiple mature teratomas (Figures 2A and 2B). No immature components were observed. Examination of the material from the rectal peritoneum revealed mature glial tissue nodules consistent with gliomatosis peritonei (Figures 2C and 2D).

The patient remained on follow-up for the next 13 months with sonographic exams every three months and monthly monitoring of serum tumor markers, which were within normal range. Contrast-enhanced thoracic and abdominal CT scans performed in July 2024 revealed no lesions suspicious for GTS, GP, or recurrence. Her serum tumor markers (AFP, CA-125, and hCG) were all normal on her final follow-up in July 2024. The patient gave written informed consent for publication of her case. Ethics Committee approval was not required by the institution for publication of this case.

## 3. Discussion

GTS is characterized by the growth in size or appearance of new metastasizing lesions containing only benign mature teratoma elements during or following chemotherapy for malignant germ cell tumors, with decreasing AFG and hCG serum tumor markers [6]. This patient met all of these criteria. The normalization of serum tumor markers occurred despite the development of new lesions, which were later confirmed as mature teratomas and glial implants through histopathological examination of the excised specimens. Theories regarding the origin of GTS suggest that it may result from the chemotherapeutic “retroconversion” of IMT elements into mature teratoma tissue. This process involves the selective destruction of malignant components while allowing the benign teratomatous elements to proliferate [7]. Another hypothesis posits that GTS could arise due to an inherent differentiation capacity of the teratomatous cells, which become evident once the malignant cells are eradicated [8].

GP is extremely rare and is seen almost exclusively with ovarian IMT. The presence of mature glial tissue implants throughout the peritoneal cavity is characteristic of GP. The incidence of GP in cases of ovarian IMT is not well documented, but it has been reported in various case studies and small series. Theories about the origin of GP suggest that the glial tissue may derive from the maturation of implanted teratomatous elements or through the metaplasia of submesothelial stem cells [9]. Genetic studies have shown that the glial cells in GP are often genetically distinct from the related teratoma, indicating that these tissues may arise independently rather than from direct metastasis of the teratoma itself [10]. These findings suggest a complex pathogenesis involving both local factors and possibly a predisposition of certain peritoneal cell populations to differentiate into glial tissue. For example, GP can be seen in the absence of a history of malignancy in patients with ventriculoperitoneal shunts, implying that it can be induced by growth factors travelling from the nervous system into the peritoneal cavity [11]. Despite its benign nature, GP has the potential for recurrence and, in rare cases, malignant transformation [12].

The coexistence of GTS and GP in patients with ovarian IMT is exceedingly rare. Several factors have been identified in the literature that may contribute to this coexistence following treatment for ovarian IMT. Residual disease and GP at the initial surgery have been noted to independently predict the occurrence of GTS [3]. Some studies have suggested that advanced stage and/or high tumor grade (2 or 3) may be predictive of GTS development in addition to incomplete resection [13]. Patients who have undergone incomplete resection of their primary IMT or who have extensive peritoneal involvement at their initial surgery have an increased likelihood of developing GTS and GP simultaneously [1,3,5,14].

The differential diagnosis of GTS and GP primarily include metastatic recurrence, immature residual tissue, and postchemotherapy necrosis or fibrosis [2,15]. Residual or recurrent malignant tissue may be associated with increasing serum tumor markers and irregular, well vascularized lesions on imaging, whereas GTS is associated with well delineated lesions with decreasing or normal serum tumor markers [8]. Additionally, malignant tissue is more likely to contain solid components [16]. Although GTS and mature teratoma may resemble each other on imaging due to the presence of cystic areas, fat, and calcifications, GTS is almost always seen following treatment, particularly after chemotherapy. Postchemotherapy necrosis or fibrosis can be visualized as masses that have not grown or are reduced in size; this is in contrast to GTS, which involves expanding lesions over time.

Peritoneal carcinomatosis can be distinguished from GP by accompanying symptoms such as ascites, as GP is generally asymptomatic. Peritoneal carcinomatosis generally consists of irregularly bordered masses and increased thickness of the peritoneum. In contrast, GP is composed of small rounded nodularities with normal peritoneal thickness and without ascites [1]. It can be challenging to discriminate GP from peritoneal tuberculosis as both may contain imaging features consisting of miliary nodules [1]. However, peritoneal tuberculosis is usually accompanied by other symptoms, such as night sweats and fever, which are absent in GP.

The time interval from the first surgery for ovarian IMT to the diagnosis of coexisting GTS and GP varies widely among patients. Typically, GTS can develop within months to several years posttreatment, with reported intervals ranging from 2 months to 60 months [1,3,5,14]. This patient’s recurrence of GTS with GP was diagnosed as early as 2 months following abdominal symptoms after the completion of chemotherapy. In many cases, the diagnosis of GTS is made within the first two years following the initial surgery, often during routine follow-up or due to symptomatic recurrence. Similarly, GP is often identified either concurrently with GTS or within a similar time frame, sometimes detected during second-look surgeries [15] or follow-up imaging. For instance, in one study [3], the median time period between initial diagnosis and the development of GTS was found to be 18.5 months, with some cases extending up to 78 months. These findings underscore the importance of prolonged and vigilant monitoring in patients with ovarian IMT as both early and late presentations of GTS and GP can occur.

The imaging features of coexisting GTS and GP in patients with ovarian IMT are characterized by distinct yet overlapping presentations of CT and magnetic resonance imaging (MRI). GTS typically manifests as enlarging masses that are poorly circumscribed, with diffuse or localized peritoneal involvement, containing prominent fatty components or cystic areas, and often showing calcifications. GP, meanwhile, presents as multiple small, smooth nodules of mature glial tissue scattered throughout the peritoneal cavity, particularly on the peritoneal surfaces and the omentum [1,3,5,14]. In the reported case, CT revealed several of these characteristic findings: the presence of a heterogeneous mass with fat and calcifications, indicative of GTS, and the detection of small nodular formations and peritoneal implants consistent with GP. It must be kept in mind that positron-emission tomography CT may show increased FDG uptake despite the absence of immature components in GTS [17].

The recurrence of GTS and GP in patients with ovarian IMT can occur at various sites within the pelvis and abdomen. Common recurrence sites for GTS include the peritoneum, retroperitoneum, and liver surfaces. Similarly, GP recurrence typically involves the peritoneal surfaces throughout the pelvic and abdominal cavity [1,3,5]. The right upper quadrant adjacent to the liver surface appears to be the most frequent site for GTS outside the pelvis [3].

The prognosis of coexisting GTS and GP in patients with ovarian IMT is generally favorable with complete surgical resection. These conditions are typically resistant to chemotherapy and radiotherapy. Surgical removal of all visible tumors may be necessary to prevent further growth and potential complications due to fistula formation, thromboembolism, mass compression, intestinal ischemia, and perforation [18–20]. Additionally, early resection of the GTS is advocated as it may harbor structural and sequential genetic abnormalities [13]. In cases of GP, the aim is to excise as many of the glial implants as possible, although some benign implants may be left in situ if they are asymptomatic and not causing complications [1,3,5,14].

Fertility-sparing surgery is of paramount importance. This approach involves preserving the uterus and at least part of one ovary while performing the necessary surgical excisions to manage the GTS and GP. The case in discussion exemplifies this approach, as the patient also underwent oocyte retrieval and cryopreservation prior to receiving further surgical treatment. There have been reports of successful pregnancy outcomes after conservative GTS treatment [3,15].

Long-term follow up of patients with GTS and GP is crucial as recurrence may be seen, especially after incomplete resection. There is also a low risk of malignant transformation associated with GP. Follow-up involves physical examination, monitoring with serum tumor markers, and imaging, particularly within the first 2 years after resection as this time period is associated with the highest risk of recurrence [1–4,15]. Imaging, in the form of CT, MRI, and ultrasound, may be used, with the latter two decreasing the risk of cumulative radiation exposure in young patients. Imaging may be performed every 3–6 months in the first 2 years and annually in the next 3 years [1–4,15]. Serum tumor markers, primarily AFP, CA-125, and hCG can be monitored every 1–3 months in the first year and every 3–6 months after that [1–4,15]. Patients should receive reproductive counselling, and pregnancies should be monitored for complications related to their conditions and previous treatment history.

The synchronous presence of GTS and GP presents unique diagnostic challenges, often mimicking disease progression or recurrence, which necessitate careful histopathological examination and long-term follow-up to ensure early detection and less aggressive treatment to manage symptomatic growths. Although they may appear to be metastatic or unresponsive to treatment, their benign nature warrants fertility-sparing surgical intervention, especially in younger women. Awareness of this condition can prevent overly aggressive surgical treatment and unnecessary use of chemotherapy.

## References

[1] Li S Su N Jia C Zhang X Yin M Growing Teratoma Syndrome with Synchronous Gliomatosis Peritonei during Chemotherapy in Ovarian Immature Teratoma: A Case Report and Literature Review Current Oncology 2022 29 9 6364 6372 10.3390/curroncol29090501 36135070 PMC9497723

[2] Bentivegna E Azaïs H Uzan C Leary A Pautier P Surgical Outcomes After Debulking Surgery for Intraabdominal Ovarian Growing Teratoma Syndrome: Analysis of 38 Cases Annals of Surgical Oncology 2015 22 3 964 970 10.1245/s10434-015-4608-y 26033179

[3] Wang D Zhu S Jia C Cao D Wu M Diagnosis and management of growing teratoma syndrome after ovarian immature teratoma: A single center experience Gynecologic Oncology 2020 157 1 94 100 10.1016/j.ygyno.2019.12.042 31954532

[4] Bonazzi C Peccatori F Colombo N Lucchini V Cantù MG Pure ovarian immature teratoma, a unique and curable disease: 10 years’ experience of 32 prospectively treated patients Obstetrics and Gynecology 1994 84 4 598 604 7522313

[5] Małgorzata SŻ Anna KG Reszeć J Krawczuk-Rybak M Growing Teratoma Syndrome and Gliomatosis Peritonei in a 15-Year-Old Girl With Immature Ovarian Teratoma: Case Report and Review of the Literature Journal of Pediatric and Adolescent Gynecology 2021 34 6 885 889 10.1016/j.jpag.2021.07.009 34314853

[6] Logothetis CJ Samuels ML Trindade A Johnson DE The growing teratoma syndrome Cancer 1982 50 8 1629 1635 10.1002/1097-0142(19821015)50:8<1629::aid-cncr2820500828>3.0.co;2-1 6288220

[7] Nitecki R Hameed N Bhosale P Shafer A Growing teratoma syndrome International Journal of Gynecologic Cancer 2023 33 2 299 303 10.1136/ijgc-2022-004265 PMC10303429 36746506

[8] Prineethi S Irodi A Eapen A Milton S Joel A Growing Teratoma Syndrome-A Clinicoradiological Series Indian Journal of Radiological Imaging 2022 2 3 301 307 10.1055/s-0042-1744519 PMC9514900 36177285

[9] Kwan MY Kalle W Lau GT Chan JK Is gliomatosis peritonei derived from the associated ovarian teratoma? Human Pathology 2004 35 6 685 688 10.1016/j.humpath.2004.01.025 15188134

[10] Gocht A Löhler J Sçheidel P Stegner HE Saeger W Gliomatosis peritonei combined with mature ovarian teratoma: immunohistochemical observations Pathology Research Practise 1995 191 10 1029 1035 10.1016/S0344-0338(11)80603-5 8838372

[11] Hill DA Dehner LP White FV Langer JC Gliomatosis peritonei as a complication of a ventriculoperitoneal shunt: case report and review of the literature Journal of Pediatric Surgery 2000 35 3 497 499 10.1016/s0022-3468(00)90221-5 10726696

[12] Yoon NR Lee JW Kim BG Bae DS Sohn I Gliomatosis peritonei is associated with frequent recurrence, but does not affect overall survival in patients with ovarian immature teratoma Virchows Archiv 2012 461 3 299 304 10.1007/s00428-012-1285-0 22820986

[13] Kikawa S Todo Y Minobe S Yamashiro K Kato H Growing teratoma syndrome of the ovary: a case report with FDG-PET findings Journal of Obstetrics and Gynaecology Research 2011 37 7 926 932 10.1111/j.1447-0756.2010.01439.x 21450035

[14] Semiz HS Sahin H Unal S Atici SD Kose MF Immature Teratoma with Growing Teratoma Syndrome and Gliomatosis Peritonei Coexistence Journal of College of Physicians and Surgeons Pakistan 2022 32 12 122 124 10.29271/jcpsp.2022.Supp0.SS122 36597312

[15] Bentivegna E Gonthier C Uzan C Genestie C Duvillard P Gliomatosis peritonei: a particular entity with specific outcomes within the growing teratoma syndrome International Journal of Gynecological Cancer 2015 25 2 244 249 10.1097/IGC.0000000000000345 25594144

[16] Han NY Sung DJ Park BJ Kim MJ Cho SB Imaging features of growing teratoma syndrome following a malignant ovarian germ cell tumor Journal of Computer Assisted Tomography 2014 38 4 551 557 10.1097/RCT.0000000000000073 24681864

[17] Hariprasad R Kumar L Janga D Kumar S Vijayaraghavan M Growing teratoma syndrome of ovary International Journal of Clinical Oncology 2008 13 1 83 87 10.1007/s10147-007-0693-7 18307026

[18] Zagamé L Pautier P Duvillard P Castaigne D Patte C Growing teratoma syndrome after ovarian germ cell tumors Obstetrics and Gynecology 2006 108 3 Pt 1 509 514 10.1097/01.AOG.0000231686.94924.41 16946208

[19] André F Fizazi K Culine S Droz J Taupin P The growing teratoma syndrome: Results of therapy and long-term follow-up of 33 patients European Journal of Cancer 2000 36 11 1389 1394 10.1016/s0959-8049(00)00137-4 10899652

[20] Merard R Ganesan R Hirschowitz L Growing Teratoma Syndrome: A Report of 2 Cases and Review of the Literature International Journal of Gynecological Pathology 2015 34 5 465 472 10.1097/PGP.0000000000000180 26262454

